# Baseline Lactate Dehydrogenase Predicts Early Treatment Failure in Diffuse Large B-Cell Lymphoma Treated With R-CHOP: A Real-World Cohort Study

**DOI:** 10.14740/jocmr6583

**Published:** 2026-06-30

**Authors:** Noorwati Sutandyo, Hilman Tadjoedin, Nia Novianti Siregar, Yuniar Harris Prayitno

**Affiliations:** a Department of Hematology and Medical Oncology, Dharmais National Cancer Center Hospital, West Jakarta, DKI Jakarta, Indonesia

**Keywords:** Diffuse large B-cell lymphoma, Early treatment failure, Primary refractory, Early relapse, Lactate dehydrogenase, R-CHOP

## Abstract

**Background:**

Early treatment failure following first-line R-CHOP (rituximab, cyclophosphamide, doxorubicin, vincristine, and prednisone) remains a major clinical problem in diffuse large B-cell lymphoma (DLBCL), particularly in real-world settings where late presentation and variable access to diagnostics may shape outcomes. We examined clinical and laboratory predictors of time-to-diagnosis associated with early treatment failure in an Indonesian referral-center cohort.

**Methods:**

We performed a retrospective observational cohort study at Dharmais National Cancer Center Hospital, Jakarta, including adults with DLBCL who initiated first-line R-CHOP between January 2019 and June 2024 and had evaluable baseline data and response assessment. Early treatment failure was defined as primary refractory disease (stable/progressive disease during or at the end of treatment, or a partial response at the end of treatment) or early recurrence occurring within ≤ 12 months following the attainment of complete response. Candidate predictors were analyzed using logistic regression; a prespecified multivariable model included age > 60 years, Eastern Cooperative Oncology Group (ECOG) performance status, Ann Arbor stage, anemia, and lactate dehydrogenase (LDH).

**Results:**

Among 372 patients diagnosed during the predefined study period, 105 with DLBCL were included in the final analytic cohort. Their mean age was 52.2 years and 54.3% were male. Early treatment failure occurred in 54/105 (51.4%): 45 (83.3%) primary refractory and nine (16.7%) early relapse. On univariate analysis, unfavorable ECOG, advanced stage, anemia, and elevated LDH were associated with early failure. In multivariable analysis, only elevated LDH remained independently associated (adjusted odds ratio (OR) 2.41, 95% confidence interval (CI) 1.01–5.79). Model discrimination was limited-to-modest (area under the curve (AUC) 0.646), indicating that baseline clinical variables alone were insufficient for strong individual-level prediction.

**Conclusion:**

In this real-world Indonesian DLBCL cohort, early treatment failure was frequent and was associated with elevated baseline LDH after adjustment. Given the limited-to-modest model discrimination and the retrospective single-center design, LDH should be interpreted as a practical warning marker for closer monitoring rather than a stand-alone predictor, and these findings require prospective validation.

## Introduction

Diffuse large B-cell lymphoma (DLBCL) is the most common aggressive subtype of non-Hodgkin lymphoma (NHL) and remains clinically challenging because outcomes vary widely despite modern therapy. In the rituximab era, first-line R-CHOP (rituximab, cyclophosphamide, doxorubicin, vincristine, and prednisone) provides long-term disease control for many patients, yet a clinically significant subgroup experiences treatment failure. Contemporary reviews consistently report that roughly 30–40% of patients are refractory to first-line therapy or relapse following a prior complete response (CR), and these early failures drive poor survival and urgent need for salvage strategies [1–4].

One of the central challenges in DLBCL care is the early identification of patients who are likely to experience early treatment failure despite standard first-line therapy. In the existing literature, early failure is generally discussed in terms of primary refractory disease and early relapse [5, 6]; however, inconsistent use of terminology has hindered comparability among studies. Recent efforts to harmonize terminology in large B-cell lymphoma have clarified that early treatment failure encompasses lack of response during or at completion of frontline immunochemotherapy, as well as relapse occurring soon after achieving CR, which is increasingly standardized as failure occurring within 12 months following completion of the first-line immunochemotherapy [7]. This framework reinforces the concept that early treatment failure represents a distinct high-risk clinical phenotype. Applying a clear and consistent time-based definition is therefore essential for evaluating candidate predictors and enabling meaningful comparisons across cohorts.

Risk stratification in DLBCL has traditionally relied on clinical prognostic indices such as the International Prognostic Index (IPI) and its refinements, which incorporate readily available factors including age, Ann Arbor stage, serum lactate dehydrogenase (LDH), performance status, and extranodal involvement. Although these indices remain valuable for broad prognostication at the population level, they were not specifically designed to predict early treatment failure in individual patients [8–10]. Furthermore, their performance and clinical utility may vary across healthcare systems with differing diagnostic access, baseline comorbidity burdens, and treatment pathways. These limitations underscore the necessity to investigate the influence of specific baseline clinical and laboratory variables—accessible at the time of diagnosis—on the likelihood of early treatment failure, especially in real-world contexts where access to advanced biological predictors may be varied.

Evidence regarding this question in low- and middle-income countries remains scarce, despite the fact that disease presentation, healthcare access, and treatment delivery may differ substantially from those reported in clinical trial populations. In Indonesia, delays in presentation, heterogeneity in diagnostic infrastructure, and context-specific host and health-system factors may shape early outcomes in ways that are not captured in Western trial cohorts. It is also necessary to define plausible, time-to-diagnosis predictors in this context to guarantee that early-failure risk classification is relevant beyond strictly regulated research settings.

To address this localized knowledge gap, the present study aimed to evaluate easily available baseline factors associated with early treatment failure in a real-world cohort of Indonesian patients with DLBCL treated with standard first-line immunochemotherapy. By identifying clinically accessible predictors tailored to the Indonesian clinical landscape, we sought to contribute evidence that may support more informed risk assessment and follow-up planning in routine practice.

## Materials and Methods

### Study design, setting, and study period

**Figure 1:**
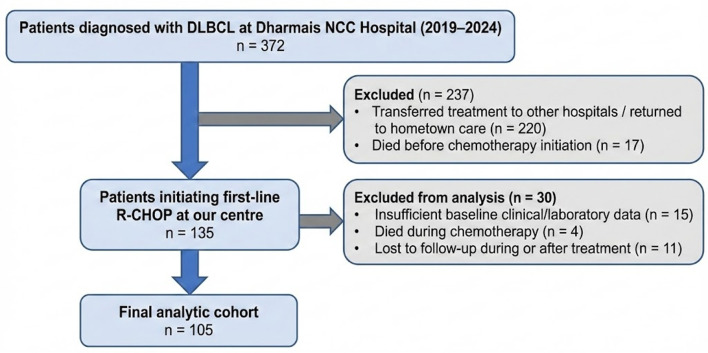
Flow diagram of patient selection and study cohort derivation. Patients diagnosed with DLBCL at Dharmais National Cancer Center Hospital between 2019 and 2024 were retrospectively screened from hospital medical records. The diagram shows sequential exclusions before cohort entry and before final analysis. The final analytic cohort consisted of 105 patients who initiated first-line R-CHOP at our center and had sufficient baseline and outcome data for early treatment failure classification. DLBCL: diffuse large B-cell lymphoma; R-CHOP: rituximab, cyclophosphamide, doxorubicin, vincristine, and prednisone.

We performed a retrospective observational cohort study at Dharmais National Cancer Center Hospital (Jakarta, Indonesia). The study population comprised consecutive patients with DLBCL who were newly diagnosed and evaluated for treatment at our center between January 2019 and June 2024. Patients included in the analytic cohort were those who initiated first-line R-CHOP at Dharmais and had sufficient baseline and response data to permit outcome classification (Fig. 1).

### Patient selection and data sources

Eligible patients were identified from hospital medical records. The cohort derivation is summarized in Figure 1. During the study period, 372 patients were diagnosed with DLBCL at Dharmais National Cancer Center Hospital. Of these, 237 patients were excluded before cohort entry because they were transferred for treatment to other hospitals or returned to hometown care (n=220), or died before chemotherapy initiation (n = 17). The remaining 135 patients initiated first-line R-CHOP at our center. Among these, 30 patients were excluded from the final analysis because of insufficient baseline clinical or laboratory data (n = 15), death during chemotherapy before adequate response assessment could be performed (n = 4), or loss to follow-up during or after treatment (n = 11). The final analytic cohort therefore consisted of 105 patients with sufficient baseline and outcome data for early treatment failure classification.

Inclusion criteria were: (1) a diagnosis of DLBCL, (2) age 18 years or older, (3) receipt of R-CHOP as first-line immunochemotherapy, and (4) availability of radiological treatment evaluation by computed tomography (CT) scan or positron emission tomography (PET)/CT, either as an interim assessment after three or four cycles of R-CHOP or after completion of treatment. Patients with insufficient documentation that hindered outcome assignment and those with double primary malignancy were excluded.

### Clinical variables and definitions

The baseline demographic characteristics encompassed age at diagnosis and sex. Age was examined as a continuous variable and pre-categorized into clinically significant strata, specifically > 60 years and < 40 years. Family history was recorded by the occurrence of cancer in first-degree relatives.

Comorbidities were determined using verified physician diagnoses made prior to or at the time of lymphoma diagnosis. The comorbid conditions evaluated included diabetes mellitus, hypertension, cardiac disease, hepatitis B, hepatitis C, chronic kidney disease (CKD), chronic obstructive pulmonary disease (COPD), and human immunodeficiency virus (HIV) infection. Overall comorbidity burden was summarized as none to one comorbidity or multiple comorbidities.

Body mass index (BMI) was determined as weight (kg) divided by height squared (m^2^). BMI was examined as a continuous variable and classified using prespecified using Asia-Pacific cut-offs.

Disease burden at presentation was characterized using Ann Arbor stage and performance status. Stage was dichotomized into limited stage (I–II) versus advanced stage (III–IV). Functional status was assessed using the Eastern Cooperative Oncology Group (ECOG) scale and analyzed as favorable (0–1) versus unfavorable (2–4).

### Baseline laboratory measurements and inflammatory indices

Baseline laboratory values were acquired from the closest available pre-treatment blood test. Anemia was evaluated as a binary variable using established hemoglobin thresholds and was defined as hemoglobin < 13 g/dL in males and < 12 g/dL in females as per WHO classification [11]. Hemoglobin was additionally summarized as a continuous measure. Serum lactate dehydrogenase (LDH) was recorded as a continuous value and categorized as elevated when the value exceeded the institutional adult upper limit of normal used by the Dharmais National Cancer Center Hospital laboratory during the study period. The institutional reference interval for LDH was ≤ 250 U/L; therefore, elevated LDH was defined as LDH > 250 U/L. This threshold is consistent with published adult chemical pathology reference intervals reporting an LDH reference interval of 120–250 U/L [12]. To evaluate systemic inflammation, we calculated the neutrophil-to-lymphocyte ratio (NLR), lymphocyte-to-monocyte ratio (LMR), and platelet-to-lymphocyte ratio (PLR) from peripheral blood counts. These indices were dichotomized using prespecified thresholds: NLR > 3, LMR < 2.5, and PLR > 150 [13–16].

### Treatment response assessment and outcome classification

All patients initiated first-line immunochemotherapy and were subsequently classified according to early treatment outcome. The primary endpoint was a composite of refractoriness and early relapse within 12 months. Treatment response was assessed using the Lugano 2014 criteria; for patients evaluated with FDG-PET/CT, metabolic response was interpreted using the Deauville 5-point score.

Primary refractory disease was defined as: (1) stable disease or progressive disease occurring during first-line chemoimmunotherapy or by end of treatment (EOT), or (2) partial response as the best response at EOT. Early relapse was defined as relapse occurring within ≤ 12 months after achieving CR at EOT. Early treatment failure was defined as primary refractory disease and early relapse (≤ 12 months) [7]. Primary refractory disease and early relapse are both classified as early treatment failure.

### Statistical analysis

All analyses were performed using IBM SPSS Statistics version 25.0 (IBM Corp., Armonk, NY, USA). Continuous variables are presented as mean ± standard deviation (SD) when approximately normally distributed and as median (interquartile range (IQR); range) when skewed; categorical variables are summarized as counts and percentages.

Baseline clinical, hematological, and inflammatory characteristics were additionally summarized according to LDH category: normal LDH (≤ 250 U/L) and high LDH (> 250 U/L). Between-group comparisons were performed using the Chi-square test or Fisher’s exact test for categorical variables, and the independent *t*-test or Mann–Whitney U test for continuous variables, as appropriate. These comparisons were considered descriptive and were used to characterize differences between LDH groups.

Predictors of early treatment failure were examined using logistic regression with a composite outcome (primary refractory and early relapse vs. sustained remission). Univariate models were fitted for each candidate predictor, reporting odds ratios (ORs) with 95% confidence intervals (CIs). Candidate predictors included age, sex, family history, comorbidity burden, BMI category, ECOG performance status, Ann Arbor stage, anemia, LDH category, and inflammatory indices (NLR, LMR, PLR).

A multivariable model was constructed using prespecified clinically relevant covariates: age > 60 years, ECOG performance status, Ann Arbor stage, anemia, and LDH category. Age, ECOG performance status, Ann Arbor stage, and LDH were chosen because they are established components of conventional DLBCL prognostic assessment, including the IPI and related prognostic frameworks [8–10]. Anemia was additionally included because it is a routinely available baseline marker that may reflect marrow involvement, systemic inflammation, nutritional status, comorbidity burden, or advanced disease biology, and has been associated with inferior outcomes in lymphoma [17–23]. The number of variables was deliberately limited in view of the modest sample size and number of early treatment failure events. Results are reported as adjusted ORs with 95% CIs for all prespecified predictors; two-sided P-values < 0.05 were considered statistically significant.

Discrimination was assessed using receiver operating characteristic (ROC) analysis and summarized as the area under the curve (AUC) with 95% CI. The ROC curve was plotted with sensitivity on the y-axis and 1-specificity on the x-axis, with a diagonal reference line representing no discrimination.

Multicollinearity was assessed using variance inflation factors (VIFs) obtained from an ordinary least squares regression of the predictor set. Model calibration was evaluated using the Hosmer–Lemeshow goodness-of-fit test and a calibration plot across deciles of predicted risk. Sensitivity analyses modeled LDH as a continuous variable (per 100 U/L increase) rather than dichotomized.

### Missing data

Missing data were handled using complete-case analysis. Patients were included in the final analytic cohort only when the baseline clinical and laboratory variables required for covariate definition and the response or follow-up data required for outcome classification were available. Therefore, there were no missing values for the variables included in the univariate or multivariable regression analyses. Patients with insufficient baseline clinical or laboratory data were excluded during cohort derivation, as shown in Figure 1. Patients who died during chemotherapy before adequate response assessment and those lost to follow-up were also excluded because early treatment failure could not be classified according to the study definition.

These exclusions may introduce selection bias. In particular, patients transferred to other hospitals, patients who died before or during chemotherapy, and patients lost to follow-up may have differed clinically from those retained in the final analytic cohort. Excluding patients who died during chemotherapy may also bias the cohort toward patients who survived long enough to undergo treatment-response assessment. Therefore, the findings should be interpreted as applying to patients with evaluable baseline and outcome data at a national tertiary referral center, rather than to all patients diagnosed with DLBCL during the study period.

### Institutional Review Board Approval

This study was reviewed and approved by the Institutional Review Board/Ethics Committee of Dharmais National Cancer Center Hospital, Jakarta, Indonesia (Approval Number: DP.04.03/11.10/68/2026).

### Ethical compliance with human/animal study

This study involved secondary data from human medical records and was conducted in accordance with institutional ethical and confidentiality requirements. All data were analyzed in de-identified form. No animal subjects were involved in this study.

## Results

### Cohort derivation

Among 372 patients diagnosed with DLBCL at Dharmais National Cancer Center Hospital between 2019 and 2024, 135 initiated first-line R-CHOP at our center. After excluding 30 patients because of insufficient baseline clinical or laboratory data, death during chemotherapy before adequate response assessment, or loss to follow-up, 105 patients were included in the final analytic cohort.

### Cohort characteristics

The final study cohort analyzed 105 patients with a confirmed diagnosis of DLBCL. Baseline clinical characteristics according to LDH category are shown in Table 1. Overall, the mean age at diagnosis was 52.2 ± 13.4 years, 28.6% of patients were older than 60 years, and 54.3% were male. Elevated LDH was present in 68 patients (64.8%), while 37 patients (35.2%) had normal LDH.

**Table 1:** Baseline Clinical Characteristics According to LDH Category

Variable	Overall cohort (n = 105)	Normal LDH ≤ 250 U/L (n = 37)	High LDH > 250 U/L (n = 68)	P value
Age at diagnosis (years)	52.2 ± 13.4	52.5 ± 13.9	52.0 ± 13.2	0.863
Age group				0.684
Below 40	19 (18.1)	8 (21.6)	11 (16.2)	
40–60	56 (53.3)	20 (54.1)	36 (52.9)	
Above 60	30 (28.6)	9 (24.3)	21 (30.9)	
Sex				0.656
Male	57 (54.3)	19 (51.4)	38 (55.9)	
Female	48 (45.7)	18 (48.6)	30 (44.1)	
Cancer in first-degree family member(s)				0.488
No	96 (91.4)	35 (94.6)	61 (89.7)	
Yes	9 (8.6)	2 (5.4)	7 (10.3)	
Diabetes mellitus				0.178
No	80 (76.2)	31 (83.8)	49 (72.1)	
Yes	25 (23.8)	6 (16.2)	19 (27.9)	
Hypertension				0.401
No	68 (64.8)	22 (59.5)	46 (67.6)	
Yes	37 (35.2)	15 (40.5)	22 (32.4)	
Cardiac disease				0.533
No	93 (88.6)	34 (91.9)	59 (86.8)	
Yes	12 (11.4)	3 (8.1)	9 (13.2)	
Hepatitis B				1.000
No	94 (89.5)	33 (89.2)	61 (89.7)	
Yes	11 (10.5)	4 (10.8)	7 (10.3)	
Hepatitis C				-
No	105 (100.0)	37 (100.0)	68 (100.0)	
Yes	0 (0.0)	0 (0.0)	0 (0.0)	
Chronic kidney disease				0.299
No	82 (78.1)	31 (83.8)	51 (75.0)	
Yes	23 (21.9)	6 (16.2)	17 (25.0)	
Chronic obstructive pulmonary disease				0.539
No	103 (98.1)	37 (100.0)	66 (97.1)	
Yes	2 (1.9)	0 (0.0)	2 (2.9)	
Human immunodeficiency virus infection				1.000
No	104 (99.0)	37 (100.0)	67 (98.5)	
Yes	1 (1.0)	0 (0.0)	1 (1.5)	
Number of comorbidities				0.110
0–1 comorbidity	72 (68.6)	29 (78.4)	43 (63.2)	
Multiple comorbidities (> 1)	33 (31.4)	8 (21.6)	25 (36.8)	
BMI (kg/m^2^)	21.8 (19.3–26.0)	21.2 (18.8–26.1)	21.9 (19.7–26.1)	0.415
BMI category				0.894
Underweight (< 18.5)	22 (21.0)	9 (24.3)	13 (19.1)	
Normoweight (18.5–22.9)	38 (36.2)	13 (35.1)	25 (36.8)	
Overweight (23.0–24.9)	13 (12.4)	5 (13.5)	8 (11.8)	
Obese (≥ 25.0)	32 (30.5)	10 (27.0)	22 (32.4)	
ECOG performance status				0.216
0	14 (13.3)	4 (10.8)	10 (14.7)	
1	61 (58.1)	25 (67.6)	36 (52.9)	
2	27 (25.7)	6 (16.2)	21 (30.9)	
3	3 (2.9)	2 (5.4)	1 (1.5)	
4	0 (0.0)	0 (0.0)	0 (0.0)	
Ann Arbor stage at diagnosis				0.241
I	12 (11.4)	6 (16.2)	6 (8.8)	
II	30 (28.6)	13 (35.1)	17 (25.0)	
III	32 (30.5)	11 (29.7)	21 (30.9)	
IV	31 (29.5)	7 (18.9)	24 (35.3)	

LDH groups were defined using the institutional upper limit of normal: normal LDH ≤ 250 U/L and high LDH > 250 U/L. Continuous variables are presented as mean ± SD or median (IQR), depending on data distribution. Categorical variables are presented as number (percentage). Between-group p values are descriptive. BMI: body mass index; ECOG: Eastern Cooperative Oncology Group; IQR: interquartile range; LDH: lactate dehydrogenase; SD: standard deviation.

A large proportion of patients (91.4%) had no first-degree relatives with cancer. Comorbid conditions were hypertension (35.2%), diabetes (23.8%), chronic renal disease (21.9%), and heart disease (11.4%). Nearly one-third of patients (31.4%) had multiple comorbidities. The median BMI was 21.8 kg/m^2^ (IQR 19.3–26.0), with 21.0% being underweight. Baseline clinical characteristics were broadly similar between the normal-LDH and high-LDH groups in descriptive comparisons.

At presentation, 60.0% of patients had advanced disease (Ann Arbor stage III–IV), and 28.6% had unfavorable ECOG performance status (2–3). Patients with high LDH numerically had more stage IV disease than those with normal LDH (35.3% vs. 18.9%), although the descriptive between-group comparison across Ann Arbor stages did not show a statistically significant difference.

### Baseline hematological and biochemical profile

Baseline hematological and inflammatory profiles according to LDH category are presented in Table 2. Overall, anemia was observed in 55.2% of patients, with a mean hemoglobin level of 12.0 ± 2.4 g/dL. The median NLR was 3.6 (IQR 2.2–7.0), with 61.9% exceeding the predefined threshold of > 3. Low LMR (< 2.5) was present in 55.2% of patients, and 73.3% had elevated PLR (> 150).

**Table 2:** Baseline Hematological and Inflammatory Profile According to LDH Category

Variable	Overall cohort (n = 105)	Normal LDH ≤ 250 U/L (n = 37)	High LDH > 250 U/L (n = 68)	P value
Hemoglobin level (g/dL)	12.0 ± 2.4	12.1 ± 2.1	11.9 ± 2.5	0.737
Anemia				0.317
Not anemic	47 (44.8)	19 (51.4)	28 (41.2)	
Anemic (male < 13; female < 12)	58 (55.2)	18 (48.6)	40 (58.8)	
Leukocyte count (× 10^3^/µL)	8.9 (5.8–11.6)	8.5 (4.9–10.6)	9.1 (6.4–11.9)	0.125
ANC (× 10^3^/µL)	5.8 (3.7–8.8)	5.3 (3.0–7.8)	6.1 (3.9–9.5)	0.073
ANC category				0.204
Low (< 1.5)	6 (5.7)	3 (8.1)	3 (4.4)	
Normal (1.5–7.0)	59 (56.2)	24 (64.9)	35 (51.5)	
High (> 7.0)	40 (38.1)	10 (27.0)	30 (44.1)	
ALC (× 10^3^/µL)	1.6 ± 0.8	1.7 ± 0.9	1.5 ± 0.8	0.343
ALC category				0.939
Low (< 1.0)	26 (24.8)	9 (24.3)	17 (25.0)	
Normal (≥ 1.0–4.8)	79 (75.2)	28 (75.7)	51 (75.0)	
NLR	3.6 (2.2–7.0)	2.9 (2.0–5.9)	4.1 (2.5–7.6)	0.042
NLR category				0.013
≤ 3	40 (38.1)	20 (54.1)	20 (29.4)	
> 3	65 (61.9)	17 (45.9)	48 (70.6)	
LMR	2.2 (1.4–3.4)	2.3 (1.6–3.8)	2.1 (1.4–3.3)	0.294
LMR category				0.555
Low (< 2.5)	58 (55.2)	19 (51.4)	39 (57.4)	
High (≥ 2.5)	47 (44.8)	18 (48.6)	29 (42.6)	
PLR	205.3 (146.5–334.3)	186.3 (126.1–281.0)	231.1 (148.5–351.4)	0.216
PLR category				0.601
Normal (≤ 150)	28 (26.7)	11 (29.7)	17 (25.0)	
High (> 150)	77 (73.3)	26 (70.3)	51 (75.0)	
Platelet count (× 10^3^/µL)	325.0 ± 123.5	321.5 ± 110.1	326.9 ± 131.0	0.822
Platelet category				0.497
Thrombocytopenia (< 150)	6 (5.7)	1 (2.7)	5 (7.4)	
Normal (150–450)	82 (78.1)	31 (83.8)	51 (75.0)	
Thrombocytosis (> 450)	17 (16.2)	5 (13.5)	12 (17.6)	

LDH groups were defined using the institutional upper limit of normal: normal LDH ≤ 250 U/L and high LDH > 250 U/L. Continuous variables are presented as mean ± SD or median (IQR), depending on data distribution. Categorical variables are presented as number (percentage). Between-group P values are descriptive. P value for LDH itself is not shown because LDH was used as the grouping variable. ALC: absolute lymphocyte count; ANC: absolute neutrophil count; LMR: lymphocyte-to-monocyte ratio; IQR: interquartile range; LDH: lactate dehydrogenase; NLR: neutrophil-to-lymphocyte ratio; PLR: platelet-to-lymphocyte ratio; SD: standard deviation.

In descriptive comparisons by LDH category, patients with high LDH had a higher median NLR than those with normal LDH (4.1 vs. 2.9; P = 0.042), and a higher proportion had NLR > 3 (70.6% vs. 45.9%; P = 0.013). Other hematological and inflammatory variables were broadly comparable between LDH groups.

### Treatment response and outcome distribution

Treatment outcomes are summarized in Table 3. Following first-line chemoimmunotherapy, 54 patients (51.4%) met the composite endpoint of early treatment failure (primary refractory disease or early relapse), while 51 patients (48.6%) maintained sustained remission across the 12-month follow-up period.

**Table 3:** Treatment Response

Treatment response	Early treatment failure (n = 54)		Stays on remission^c^ (n = 51)
	Primary refractory disease^a^ (n = 45)	Early relapse^b^ (n = 9)	
CR	-	9 (100)	51 (100)
PR	22 (48.8)	-	-
SD	6 (13.4)	-	-
PD	17 (37.8)	-	-

^a^Primary refractory disease was defined as: (1) stable disease or progressive disease occurring during first-line chemoimmunotherapy or by EOT, or (2) partial response as the best response at EOT. ^b^Early relapse was defined as relapse occurring within ≤ 12 months after achieving CR at EOT. ^c^Patients classified as “stays on remission” achieved CR and had no evidence of relapse during follow-up. Patients without a failure event were administratively censored at 12 months. Data are presented as n (%) for categorical variables. CR: complete response; EOT: end of treatment; PD: progressive disease; PR: partial response; SD: stable disease.

Within the early treatment failure group (54 patients), 45 patients (83.3%) were classified as primary refractory disease, and nine (16.7%) experienced early relapse within ≤ 12 months after achieving CR at EOT. Among primary refractory cases, best response consisted of partial response in 22 (48.8%), stable disease in six (13.4%), and progressive disease in 17 (37.8%). The remaining 51 patients who stayed on remission achieved CR with no evidence of relapse during follow-up and were classified as sustained remission at 12 months.

### Univariate predictors of refractoriness or early relapse

The results of the univariate logistic regression analysis are presented in Table 4. Several established markers of tumor burden and biological aggressiveness were significantly associated with refractoriness.

**Table 4:** Univariate Analysis

Variable	Outcome		OR (95% CI)	P value
	Stay on remission	Early treatment failure (refractory and early relapse)		
Age (years)				
≤ 60	36 (48.0)	39 (52.0)	Ref	Ref
> 60	15 (50.0)	15 (50.0)	0.923 (0.396–2.153)	0.853
Sex				
Male	28 (49.1)	29 (50.9)	Ref	Ref
Female	23 (47.9)	25 (52.1)	1.049 (0.487–2.263)	0.902
Cancer history in first-degree family				
No	45 (46.9)	51 (53.1)	Ref	Ref
Yes	6 (66.7)	3 (33.3)	0.441 (0.104–1.867)	0.266
Diabetes mellitus				
No	40 (50.0)	40 (50.0)	Ref	Ref
Yes	11 (44.0)	14 (56.0)	1.273 (0.516–3.140)	0.601
Hypertension				
No	30 (44.1)	38 (55.9)	Ref	Ref
Yes	21 (56.8)	16 (43.2)	0.602 (0.268–1.349)	0.217
Heart disease				
No	45 (48.4)	48 (51.6)	Ref	Ref
Yes	6 (50.0)	6 (50.0)	0.938 (0.282–3.120)	0.916
Hepatitis B				
No	48 (51.1)	46 (48.9)	Ref	Ref
Yes	3 (27.3)	8 (72.7)	2.783 (0.695–11.140)	0.148
CKD				
No	41 (50.0)	41 (50.0)	Ref	Ref
Yes	10 (43.5)	13 (56.5)	1.300 (0.512–3.299)	0.581
Comorbidity				
0–1 comorbidity	34 (47.2)	38 (52.8)	Ref	Ref
Multiple comorbidities (> 1)	17 (51.5)	16 (48.5)	0.842 (0.369–1.921)	0.683
BMI (kg/m^2^)				
Underweight (<18.5)	8 (36.4)	14 (63.6)	Ref	Ref
Not underweight (≥18.5)	43 (51.8)	40 (48.2)	0.532 (0.202–1.402)	0.201
ECOG score				
Favorable ECOG (0–1)	42 (56.0)	33 (44.0)	Ref	Ref
Unfavorable ECOG (2–4)	9 (30.0)	21 (70.0)	2.970 (1.202–7.335)	0.018*
Stage at diagnosis				
Limited-stage (I–II)	26 (61.9)	16 (38.1)	Ref	Ref
Advanced-stage (III–IV)	25 (39.7)	38 (60.3)	2.470 (1.108–5.506)	0.027*
Hemoglobin level (g/dL)				
Not anemic	28 (59.6)	19 (40.4)	Ref	Ref
Anemic (male < 13; female < 12)	23 (39.7)	35 (60.3)	2.243 (1.023–4.916)	0.044*
ANC				
Low–normal (≤ 7)	35 (53.8)	30 (46.2)	Ref	Ref
High (> 7)	16 (40.0)	24 (60.0)	1.750 (0.787–3.890)	0.170
ALC				
Low (< 1)	10 (38.5)	16 (61.5)	Ref	Ref
Normal (≥ 1–4.8)	41 (51.9)	38 (48.1)	0.579 (0.234–1.432)	0.237
NLR				
≤ 3	22 (55.0)	18 (45.0)	Ref	Ref
> 3	29 (44.6)	36 (55.4)	1.517 (0.687–3.350)	0.302
LMR				
Low (< 2.5)	25 (43.1)	33 (56.9)	Ref	Ref
High (≥ 2.5)	26 (55.3)	21 (44.7)	0.612 (0.282–1.328)	0.214
PLR				
Normal (≤ 150)	13 (46.4)	15 (53.6)	Ref	Ref
High (> 150)	38 (49.4)	39 (50.6)	0.889 (0.374–2.116)	0.791
Platelet count				
Low to normal (≤ 450)	43 (48.9)	45 (51.1)	Ref	Ref
Thrombocytosis (> 450)	8 (47.1)	9 (52.9)	1.075 (0.380–3.042)	0.892
LDH				
Normal (≤ 250)	24 (64.9)	13 (35.1)	Ref	Ref
High (> 250)	27 (39.7)	41 (60.3)	2.803 (1.220–6.439)	0.015*

*Statistically significant. ALC: absolute lymphocyte count; ANC: absolute neutrophil count; BMI: body mass index; CI: confidence interval; CKD: chronic kidney disease; COPD: chronic obstructive pulmonary disease; ECOG: Eastern Cooperative Oncology Group; HIV: human immunodeficiency virus; LDH: lactate dehydrogenase; LMR: lymphocyte to monocyte ratio; NLR: neutrophil to lymphocyte ratio; OR: odds ratio; PLR: platelet to lymphocyte ratio.

Unfavorable ECOG performance status (2–3 vs. 0–1) was associated with nearly a threefold increased odds of refractory disease/early relapse (OR 2.970, 95% CI 1.202–7.335; P = 0.018). Advanced stage (III–IV vs. I–II) conferred a 2.47-fold higher odds (OR 2.470, 95% CI 1.108–5.506; P = 0.027). Anemia was associated with a 2.24-fold increase in chances (OR 2.243, 95% CI 1.023–4.916; P = 0.044). Elevated LDH showed the strongest univariate association, with a 2.80-fold increased risk (OR 2.803, 95% CI 1.220–6.439; P = 0.015).

Conversely, age > 60 years, sex, BMI category, comorbidity burden, and inflammatory indicators (NLR, LMR, PLR) had no statistically significant association with the composite outcome.

### Multivariable analysis

Adjusted ORs with 95% CIs for all prespecified predictors are presented in Table 5. After adjustment for age, ECOG performance status, Ann Arbor stage, anemia, and LDH level, elevated LDH remained independently associated with refractoriness or early relapse (adjusted OR 2.412, 95% CI 1.006–5.785; P = 0.048).

**Table 5:** Multivariable Logistic Regression Analysis of All Prespecified Predictors for Early Treatment Failure

Variable	Adjusted OR (95% CI)	P value
Unfavorable ECOG (vs. 0–1)	2.228 (0.846–5.869)	0.105
Advanced stage III–IV (vs. I–II)	1.843 (0.782–4.341)	0.162
Anemia (vs. not anemia)	1.611 (0.684–3.797)	0.276
High LDH (vs. normal)	2.412 (1.006–5.785)	0.048*
Age > 60 (vs. ≤ 60)	0.888 (0.357–2.209)	0.799

*Statistically significant. Adjusted odds ratios were derived from a prespecified multivariable logistic regression model including age > 60 years, ECOG performance status, Ann Arbor stage, anemia, and LDH category. CI: confidence interval; ECOG: Eastern Cooperative Oncology Group; LDH: lactate dehydrogenase; OR: odds ratio.

The associations observed for ECOG performance status (adjusted OR 2.228; P = 0.105), advanced stage (adjusted OR 1.843; P = 0.162), and anemia (adjusted OR 1.611; P = 0.276) were attenuated and no longer statistically significant after adjustment. Age older than 60 years was not associated with outcome (adjusted OR 0.888; P = 0.799).

Multicollinearity was not evident (all VIFs approximately 1.0–1.1).

### Model discrimination

**Figure 2:**
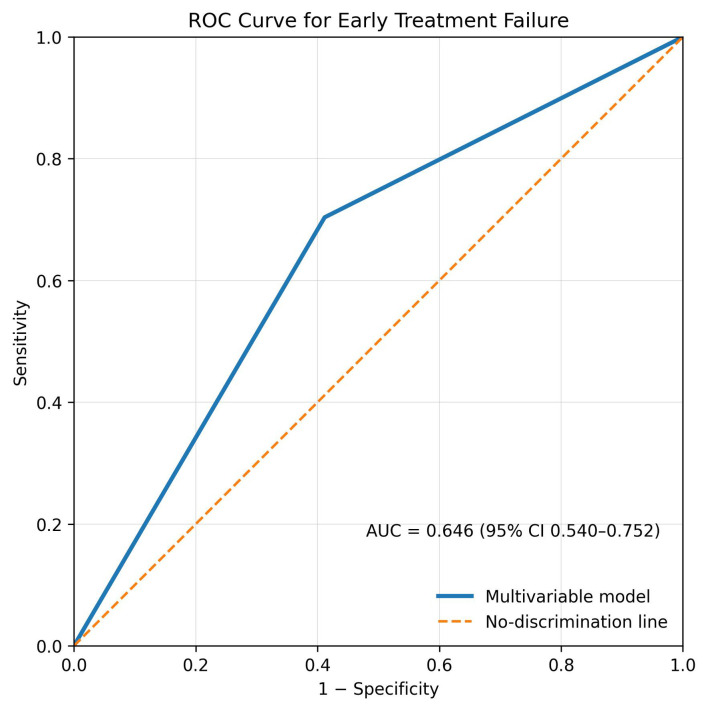
Discriminative performance of the multivariable logistic regression model for ETF in DLBCL (AUC = 0.646; 95% CI 0.540–0.752). The ROC curve demonstrates limited-to-modest discriminative ability of the model incorporating ECOG performance status, disease stage, anemia, LDH level, and age group. The AUC was 0.646 (95% CI 0.540–0.752; P = 0.010). AUC: area under the curve; CI: confidence interval; DLBCL: diffuse large B-cell lymphoma; ECOG: Eastern Cooperative Oncology Group; ETF: early treatment failure; LDH: lactate dehydrogenase; ROC: receiver operating characteristic.

Model discrimination is shown in Figure 2. The model demonstrated limited-to-modest discriminatory performance (AUC 0.646, 95% CI 0.540–0.752; P = 0.010). This indicates performance above chance, but not sufficient accuracy for use as a stand-alone individual-level prediction model.

### Model calibration

Model calibration was assessed using the Hosmer–Lemeshow goodness-of-fit test (χ^2^ = 5.175, df = 8, P = 0.739) and a calibration plot across deciles of predicted risk, which showed broadly similar observed and predicted event rates.

## Discussion

In this real-world Indonesian cohort of 105 patients with DLBCL treated with first-line R-CHOP, early treatment failure was prevalent: approximately 50% of patients met the composite endpoint of refractory disease or early relapse. In this clinical context, we discovered that several routinely available baseline factors—unfavorable ECOG performance status, advanced stage, anemia, and elevated LDH—were associated with early failure on univariate analysis, but only LDH remained independently associated with the composite outcome after adjustment. The resulting multivariable model demonstrated statistically significant yet modest discrimination (AUC 0.646), highlighting both the benefit and limitations of using easily available clinical factors for individual-level prediction.

### Understanding early failure in a referral-center cohort

The proportion of patients experiencing early treatment failure in our cohort appears higher than similar reports from trial populations and some large observational series [24–26], and the pattern of failure—refractory disease with a short time-to-event—suggests a clinically aggressive subgroup enriched in routine practice at a national referral center. Several contextual factors could plausibly contribute. First, advanced stage at diagnosis was frequent, and LDH elevation—often a surrogate of tumor burden, rapid proliferation, and tissue destruction [27, 28]—was present in nearly two-thirds of patients, consistent with biologically aggressive disease at presentation. Second, real-world diagnostic and delivery of immunochemotherapy can be influenced by delayed presentation, heterogeneous diagnostic access, comorbidity constraints, and treatment interruptions, all of which may be more prominent in low- and middle-income settings than in clinical trials. Third, selection effects are likely: tertiary centers often manage a higher-risk case-mix, including patients referred late, with bulky disease, poor performance status, or complicated medical backgrounds. These considerations do not diminish the value of our findings; rather, they make them particularly relevant to everyday decision making in comparable settings, where clinicians must identify high-risk patients early using information that is available on day 1.

The study period also overlapped with the COVID-19 pandemic, which may have influenced patient presentation, diagnostic timelines, and treatment delivery. International evidence has shown that the pandemic disrupted cancer services through delays in diagnosis, treatment initiation, follow-up, and general oncology care [29]. At Dharmais National Cancer Center Hospital, cancer patients with COVID-19 represented a clinically vulnerable group, and delayed cancer treatment was identified among factors associated with poor outcomes [30]. For aggressive lymphomas, however, expert guidance during the pandemic emphasized that newly diagnosed or relapsing aggressive lymphoma should generally continue to be treated according to standard guidelines, as broad treatment deferral may compromise curative intent [31]. Therefore, the high rate of early treatment failure in our cohort may partly reflect pandemic-era disruption, including delayed presentation, delayed referral, delayed treatment initiation, treatment interruption, or incomplete follow-up. However, the present study could not quantify the direct impact of COVID-19 because symptom-onset dates, referral intervals, SARS-CoV-2 infection status, chemotherapy dose intensity, and treatment-delay duration were not uniformly available. This should be considered a contextual limitation when interpreting the findings.

### LDH as the main independent predictor

The key finding of our multivariable analysis was that elevated LDH remained independently associated with early treatment failure (adjusted OR 2.41), whereas ECOG, stage, and anemia attenuated and crossed conventional significance thresholds. Clinically, this result is clear. LDH is not merely “another lab test;” it is a condensed signal that captures numerous biological and anatomical characteristics of aggressive lymphoma: extensive tumor burden, high tumor proliferation, and high tumor metabolic activity [17, 18, 32]. Elevated LDH in aggressive lymphomas largely reflects increased glycolytic metabolism and rapid cellular turnover, processes that accompany highly proliferative tumor clones and increased tumor cell destruction. It is also one of the foundational components of prognostic indices used in DLBCL, reflecting decades of consistent association with inferior outcomes across diverse cohorts.

Statistically, LDH may serve as a surrogate for several associated factors [33]. Performance status, stage, and anemia are frequently interconnected with tumor burden and systemic inflammation [19, 20]. For example, patients with extensive disease burden or more aggressive tumor biology may simultaneously present with elevated LDH, poorer functional status, and anemia related to marrow involvement, chronic inflammation, or treatment-limiting comorbidities. In a moderately sized dataset, multicollinearity and shared explanatory variance may result in the reduction of effects when variables are combined, while each exhibiting a distinct univariate correlation. This does not mean that ECOG, stage, or anemia are unimportant. Rather, in this cohort, their prognostic value may substantially overlap with LDH, which could explain why they did not appear as independent predictors when analyzed together.

From a practical standpoint, the persistence of LDH after adjustment suggests its value as a readily available warning marker for early risk conversations and follow-up planning when more granular biological risk stratification, such as cell of origin, double expressor status, cytogenetics, or ctDNA, is not routinely available. This interpretation should remain cautious, as the model showed limited-to-modest discrimination and requires prospective validation before being used for individual-level risk prediction.

### ECOG, stage, and anemia: useful markers in the real world

In univariate analysis, unfavorable ECOG and advanced stage were individually linked to probabilities of early failure that were about 2.5 to 3 times greater, and anemia doubled the odds. These findings are consistent with established clinical observations: frailer patients exhibit reduced treatment tolerance [3], advanced-stage disease signifies increased dissemination [21], and baseline anemia may indicate marrow involvement, systemic inflammation, nutritional deficiency, or chronic disease [20, 34–36], each potentially associated with inferior outcomes. Notably, 16 of the 42 individuals in our cohort with limited-stage lymphoma (38.1%) also experienced early failure. This also reveals that early-stage lymphoma may still have adverse biological traits, like high proliferative activity, aggressive molecular subtypes, or high tumor metabolic activity [22, 23, 37].

There is also a clinical distinction that merits emphasis. ECOG performance status can be both a confounder and a mediator: it may reflect disease burden (patients are unwell because their lymphoma is advanced), but it can also influence treatment intensity, dose reductions, delays, and supportive care needs. In settings where treatment delivery is variably constrained, performance status may have a stronger relationship with outcomes than in tightly controlled trials. Similarly, anemia may be more multifactorial in real-world populations [20], where co-existing nutritional anemia, chronic infection/inflammation, and delayed diagnosis are more common. In these circumstances, anemia may serve as a composite indicator of both host susceptibility and disease pathology [38, 39], even if it does not appear as “independently significant” in a model primarily influenced by LDH.

### Inflammatory ratios: why they fell short in our cohort

The LDH-stratified baseline comparison showed that patients with high LDH had higher NLR and a higher proportion of NLR > 3 than those with normal LDH, suggesting some overlap between LDH elevation and systemic inflammatory burden. However, despite this pattern, none of the inflammatory indices was statistically associated with early treatment failure in the outcome analysis. This negative finding is informative. Peripheral blood ratios are attractive because they are cheap and widely available, but their prognostic value may be sensitive to timing, intercurrent infection, steroid exposure, and unmeasured confounders. [40, 41]. In Indonesia, where concurrent infections, inflammatory comorbidities, and variability in pre-diagnostic care are relatively common, these ratios may capture both tumor-related and non-tumor-related inflammatory processes [42]. In the context of early treatment failure—an outcome closely linked to tumor burden and intrinsic disease aggressiveness—systemic inflammatory indices may offer limited incremental discrimination once LDH and other burden-related markers are incorporated into multivariable models. The implication is not that inflammatory indices are useless, but that—at least in this cohort—they did not improve the practical day-1 prediction of early failure.

### What limited discriminatory performance means in practice

An AUC of 0.646 indicates that the model discriminated early treatment failure from sustained remission better than chance, but with limited discriminatory performance. This interpretation is deliberately cautious, as classic methodological literature describes AUC values between approximately 0.50 and 0.70 as representing low accuracy [43]. Moreover, AUC reflects discrimination only and does not, by itself, establish clinical utility, because it does not account for the consequences of false-positive and false-negative risk classification [44, 45]. Therefore, LDH should be interpreted as a practical risk marker rather than a stand-alone prediction tool. Elevated LDH may help identify patients who require closer surveillance, careful attention to treatment delivery, and timely response assessment, but the observed AUC does not support using LDH, either alone or within this simple baseline model, to alter first-line treatment decisions. The attenuation of established prognostic factors, including ECOG performance status, Ann Arbor stage, and anemia, after adjustment may reflect overlapping prognostic information among baseline markers of tumor burden, metabolic activity, systemic inflammation, and host condition. Patients with extensive or biologically aggressive lymphoma may simultaneously present with elevated LDH, functional decline, advanced-stage disease, and anemia related to marrow involvement, inflammation, nutritional compromise, or chronic disease. Consequently, these variables may show significant associations in univariate analysis but lose independent significance when entered together in a moderately sized multivariable model. This does not mean that ECOG performance status, disease stage, or anemia is clinically unimportant. Rather, in this cohort, much of the prognostic information carried by these variables may have overlapped with LDH, leaving limited additional predictive value after adjustment. Initial treatment failure in DLBCL is not exclusively dictated by baseline clinical severity, as outcomes are also influenced by molecular subtype, high-risk biological traits including double-hit/double-expressor status and TP53 alterations, therapy intensity and delivery, and early response dynamics [46–50]. Together, these findings support the need for enhanced risk-stratification approaches that combine baseline clinical variables with tumor biology and dynamic response markers, including primary refractory disease frameworks, event-free survival at 24 months (EFS24), and prognostic models specifically designed to anticipate early chemoimmunotherapy failure.

### Clinical implications for Indonesian practice

In routine care, clinicians rarely have immediate access to the full spectrum of biological risk markers at diagnosis. Our findings suggest that elevated LDH, interpreted together with the broader clinical context rather than in isolation, may serve as an early warning signal that first-line R-CHOP may not translate into durable disease control. Rather than changing therapy empirically, the actionable consequence is intensified delivery: meticulous supportive care to avoid dose delays, heightened vigilance for non-response during treatment, and a faster transition to re-staging and salvage planning when the clinical course is not clearly favorable.

At a service level, the high proportion of early failure also points to potential system opportunities: reducing diagnostic delay, standardizing staging and response assessment where possible, and strengthening supportive care pathways (infection prophylaxis where appropriate, nutritional optimization, management of anemia and comorbidities) to maintain chemotherapy dose intensity. Even modest improvements in timely presentation and treatment delivery could translate into meaningful gains in early outcomes, particularly for patients who are borderline fit at baseline.

### Strengths and limitations

This study’s strengths lie in its real-world design, clearly defined composite endpoint within a clinically meaningful 12-month window, and focus on variables that are routinely available in everyday care. These features increase the applicability of the findings to similar centers and health systems.

Several limitations merit emphasis. First, the retrospective single-center design introduces selection bias and limits generalizability beyond comparable referral settings. Second, the sample size constrains multivariable modeling and reduces precision, particularly for variables with modest effects or low prevalence. Third, we were unable to incorporate key biological predictors (cell-of-origin classification, double expressor/double hit status, cytogenetics, and other genomic markers) that are strongly linked to refractory disease and would likely improve model performance. Fourth, treatment delivery factors (dose reductions, delays, completion rates) and dynamic response measures were not analyzed here but may be critical determinants of early failure. In addition, because the study period overlapped with the COVID-19 pandemic, unmeasured pandemic-related effects on presentation, referral pathways, diagnostic timing, treatment delivery, and follow-up may have influenced the observed rate of early treatment failure. Finally, our endpoint was confined to a 12-month period; while appropriate for capturing early aggressive behavior, it does not address late relapses or long-term survival outcomes, and it should be viewed as complementary to longer-term endpoints such as EFS24, and overall survival.

### Future directions

Future work should aim to (1) validate these findings in a multicenter Indonesian cohort to reduce center-specific bias; (2) integrate accessible tumor biology (at minimum, cell of origin and double expressor status where feasible); and (3) evaluate whether adding dynamic markers—early radiological response or ctDNA kinetics—improves discrimination for early failure beyond baseline LDH. Parallel work could explore pragmatic, context-adapted prediction tools that balance feasibility with performance, rather than importing models developed in settings with broader biomarker availability. Recent efforts to create indices specifically for early chemoimmunotherapy failure highlight the direction of travel: the clinical question is shifting from “overall prognosis” to “who is going to fail early on R-CHOP?”, and answering that question will likely require a hybrid of baseline clinical severity plus biological and dynamic response data.

### Conclusion

In summary, early treatment failure within 12 months was frequent in this Indonesian real-world DLBCL cohort, with events dominated by primary refractory disease. Among routinely available baseline factors, elevated LDH showed the strongest adjusted association with early treatment failure, while ECOG performance status, stage, and anemia showed meaningful univariate associations. However, the limited-to-modest model discrimination and the retrospective single-center design mean that LDH should not be interpreted as a stand-alone prediction tool or as a basis for altering first-line treatment decisions. Rather, LDH may serve as a practical warning marker to support closer monitoring, timely response assessment, and early planning when the clinical course is unfavorable. Prospective multicenter validation is needed to confirm these findings and to determine whether combining LDH with tumor biology, treatment-delivery variables, and dynamic response measures can improve prediction of early failure.

## Data Availability

The data supporting the findings of this study are available from the corresponding author upon reasonable request.
